# Report of Two Cases—One of Alveolar Abscess, and the Other of the Injury of the Inferior Maxillary

**Published:** 1852-01

**Authors:** Charles Bew

**Affiliations:** Surgeon Dentist to His late Majesty William the Fourth.


### ARTICLE VII.

*Report of Two Cases—one of Alveolar Abscess, and the other of Injury of the Inferior Maxillary.* By Charles Bew, Surgeon Dentist to His late Majesty William the Fourth.

Case First. Soon after my residence in Brook street, London, a young man, one of the messengers of the Lord Chamberlain's office, had been for some time suffering from the weeping of a formidable abscess, beneath the angle of the left side of the under jaw, which discharged pus of a viscid and fetid character as copiously as uncomfortably. The young man called on me, with a note from the surgeon of the household, which stated the young man's ailment to arise from a portion of a diseased dens sapientiæ in the left under jaw. On examining the patient, I found him pale, much reduced, and altogether a prey to misery of no ordinary degree, and much apprehension, having been told that unless the diseased tooth could be extracted, his life would be placed in peril through accession of fever. I comforted and encouraged the poor sufferer, all in my power, and commenced my examination. The crown of the tooth had disappeared, whether by disease or fracture, he could not recollect, but said he thought it had been taken out in consequence of aching, that is, the cap broke off and the root left in the alveolus. The stump or remaining portion of the root, I could but very indistinctly see from the swollen state of the gum on the coronoid process, obscured as it was by a molar tooth. But I could pass the nail of my thumb and right index finger all around the stump, which encouraged me to hope I should be able to fix a peculiarly shaped lever key, bent into the form of the letter S, to it, and by which, while my hand was high above the associated side teeth, I found I could get a firm low hold on the stump itself. I first moved, and then raised it upwards in a small degree, perceptible by the discharge of black clotted venous blood which followed; but as this was effected with considerable pain to the patient and fatigue to myself, the mouth was rinsed with warm water as long as the blood continued to flow, when I saturated some lint with a warm spiritous tincture, which left him at comparative ease. I made an appointment with him for the next morning, my first attempt having been made in the afternoon, being well aware that the ordinary contraction of the alveolus on the conical root, would be sufficient to raise it enough by morning to enable me to extract it with certainty. The secretion of pus during the night on the conical root made it feel as though it had been soaped, and when out, it was difficult to secure it between the thumb and finger. The removal of the painful cause, produced the desired effect. My patient was at ease and a stop put to the inflammatory action which had been going on so long, with its concomitant formation of the pus or acrid matter. This having no other way of escape, made for itself a passage through the socket and jaw-bone, discharging itself on his shirt collar, and a pledget of lint placed under his chin and secured by means of a silk handkerchief tied over his head. The abscess disappeared in a week, and he was soon restored to sound health.

CASE SECOND. D. W. Keoghan, formerly one of the carters to Barclay's distillery, South Lambreth, on my taking possession of the house I now reside in, a man who looks after a Protestant Chapel, of which my landlord is the curate, had been recommended to see the putting in of my furniture, and in the course of his attendance, requested me to look at, and give my opinion upon the sad state of his teeth and mouth. I found his mouth in a dreadful state of inflammation, with exfoliation of the under jaw, and the four incisors ready to drop out. I observed to him, that he appeared to have had a very serious accident, and may I ask how it happened? One day in descending from the cart, some one called to him while in the act of jumping from it, and by diverting his attention, his under jaw caught against the tail-board, he was stunned and fell insensible in the road in which the cart was standing. He was confined to his bed six weeks, and when he was able to come out to his work, his suffering was scarcely supportable. On my first seeing him, he asked if it were possible to have a cure effected? I encouraged him to believe that, with care and attention, he might be restored, and that as soon as I was settled, I would attend to his case, which I accordingly did.

The four incisors were so loose, that they were moved by every touch of the tongue, and more than half of the under jaw was in a frightful state of exfoliation. Two abscesses were discharging large quantities of purulent matter, which, together with the constitutional effects of the local irritation, and the difficulty he experienced in taking food, threatened to run the poor sufferer very soon into his grave. Under these circumstances, the four incisors were first removed, and then commenced the work of taking away the exfoliating bone. This operation was long and tedious. Five pieces, as black as ebony, were in as many weeks taken away, relieving the poor man of pain and fetor almost unbearable. This accomplished, the parts healed rapidly, and by the kindness of one of my mechanical friends, the incisors were neatly placed in a metallic *Ratelier* and so fitted to the mouth, that no sign is at present discernible, either of his sufferings or disfigurement. He takes his food without difficulty, and is in better health than he has been for years.

**15 South Lambreth, near the Vauxhall Bridge, Railway Station.**
*October* 16, 1851.

